# Systematic Study of Ferromagnetism in Cr_x_Sb_2−x_Te_3_ Topological Insulator Thin Films using Electrical and Optical Techniques

**DOI:** 10.1038/s41598-018-35118-8

**Published:** 2018-11-19

**Authors:** Angadjit Singh, Varun S. Kamboj, Jieyi Liu, Justin Llandro, Liam B. Duffy, Satyaprasad P. Senanayak, Harvey E. Beere, Adrian Ionescu, David A. Ritchie, Thorsten Hesjedal, Crispin H. W. Barnes

**Affiliations:** 1 Cavendish Laboratory, University of Cambridge, J. J. Thomson Avenue, Cambridge, CB3 0HE United Kingdom; 2 Clarendon Laboratory, Department of Physics, University of Oxford, Oxford, OX1 3PU United Kingdom; 3 Laboratory for Nanoelectronics and Spintronics, Research Institute of Electrical Communication, Tohoku University, 2-1-1 Katahira, Aoba-ku, Sendai, 980-8577 Japan; 4 ISIS, Rutherford Appleton Laboratory, Harwell Science and Innovation Campus, Science and Technology Facilities Council, Oxon, OX11 0QX United Kingdom; 5 Laboratory for Advanced Research in Polymeric Materials (LARPM), Central Institute of Plastics Engineering and Technology (CIPET), B-25, CNI complex, Patia, Bhubaneswar, Odisha, 751024 India

## Abstract

Ferromagnetic ordering in a topological insulator can break time-reversal symmetry, realizing dissipationless electronic states in the absence of a magnetic field. The control of the magnetic state is of great importance for future device applications. We provide a detailed systematic study of the magnetic state in highly doped Cr_x_Sb_2−x_Te_3_ thin films using electrical transport, magneto-optic Kerr effect measurements and terahertz time domain spectroscopy, and also report an efficient electric gating of ferromagnetic order using the electrolyte ionic liquid [DEME][TFSI]. Upon increasing the Cr concentration from *x* = 0.15 to 0.76, the Curie temperature (*T*_c_) was observed to increase by ~5 times to 176 K. In addition, it was possible to modify the magnetic moment by up to 50% with a gate bias variation of just ±3 V, which corresponds to an increase in carrier density by 50%. Further analysis on a sample with *x* = 0.76 exhibits a clear insulator-metal transition at *T*_*c*_, indicating the consistency between the electrical and optical measurements. The direct correlation obtained between the carrier density and ferromagnetism - in both electrostatic and chemical doping - using optical and electrical means strongly suggests a carrier-mediated Ruderman-Kittel-Kasuya-Yoshida (RKKY) coupling scenario. Our low-voltage means of manipulating ferromagnetism, and consistency in optical and electrical measurements provides a way to realize exotic quantum states for spintronic and low energy magneto-electronic device applications.

## Introduction

The term “topological insulator” (TI) was first introduced in 2007 to generalize the two-dimensional quantum spin Hall state to three dimensions^1,2^. The key reasons were the presence of time reversal symmetry (TRS) and spin-momentum locking^3,4^. Since then, researchers have been in search of exotic quantum phenomena which can potentially revolutionize the field of condensed matter physics. Transition metal doping in TIs is predicted to break TRS which opens a surface gap at the Dirac point^5,6^. This has led to the recent experimental observations of the topological magnetoelectric effect^7^, induction of a magnetic monopole^8^ and the quantum anomalous Hall effect (QAHE)^9^. Hence a wide range of potential industrial applications have found their way into the literature such as magnetic sensing^10^, information storage^10^, phase change memories^11^ and various spin injectors for spintronic devices^12^. In the quantized version of the anomalous Hall effect (AHE), the topologically protected state has a dissipationless current flowing along the edge of a 2D surface of a magnetically doped TI in the absence of a magnetic field. In this context, Cr-doped Sb_2_Te_3_ has been the object of consideration since the discovery that it becomes ferromagnetic when Cr replaces Sb^13^. The AHE is a well-recognized phenomenon and has been extensively studied in transition metal doped dilute magnetic semiconductors (DMS) and complex oxide ferromagnets^14^. To ascertain the general origin of the AHE, various theories have also been put forward^14^. In TIs, on the other hand, it is now well established that the origin relies on a scattering-independent, intrinsic mechanism which can be described in terms of the Berry phase effect in crystal momentum space^9,14–16^. The evolution of the AHE with temperature and magnetic field can be linked to the magnetization, *M*, of the sample through the following relationship^14^: *ρ*_*xy*_ = *R*_*o*_*B* + *R*_*s*_*M*, where *ρ*_*xy*_ is the total Hall resistivity, *B* is the applied magnetic field, *R*_*o*_ = 1/*nec* (*e* being the electron charge and *n* the carrier concentration) is the ordinary Hall coefficient arising from the Lorentz force experienced by electrons/holes, and *R*_*s*_ is the anomalous Hall coefficient. On dividing this equation by ${\rho }_{xx}^{2}$, we see that the equation expresses the additivity of the Hall currents, that is, the total Hall conductivity ${\sigma }_{xy}^{T}$. As ${\rho }_{xy}\ll {\rho }_{xx}$, ${\sigma }_{xy}^{T}=\frac{{\rho }_{xy}}{{\rho }_{xx}^{2}+\,{\rho }_{xy}^{2}}\approx \frac{{\rho }_{xy}}{{\rho }_{xx}^{2}}=\frac{RoB+\,{R}_{s}M}{{\rho }_{xx}^{2}}$ with ${\sigma }_{xy}^{T}={\sigma }_{xy}^{O}+{\sigma }_{xy}^{A}$, where ${\sigma }_{xy}^{O}$ and ${\sigma }_{xy}^{A}$ are the ordinary and anomalous Hall conductivity respectively. These result in: ${\sigma }_{xy}^{A}={\sigma }_{xy}^{T}-{\sigma }_{xy}^{O}=\frac{{R}_{s}M}{{\rho }_{xx}^{2}}$. From this equation, an accurate determination of ${\sigma }_{xy}^{A}$ will indirectly govern the magnetisation *M* by carefully removing the ordinary Hall component ${\sigma }_{xy}^{O}=\frac{{R}_{O}B}{{\rho }_{xx}^{2}}$, hence enabling the field and temperature dependence in *M* to be extracted directly from magnetotransport data. Given this context, there is also a strong motivation to understand the mechanism of the magnetic behaviour in TIs. Two models have been proposed in the literature: the Van Vleck mechanism^17^ which is caused by the large spin susceptibility of the valence electrons in the band-inverted TI materials and the Rudderman-Kittel-Kasuya-Yosida (RKKY)^5^ coupling, where the magnetic ions can indirectly couple with the assistance of itinerant electrons. Consequently, the former is independent of the carrier density and is referred as “bulk ferromagnetism”, while in the latter case the neighbouring magnetic moments couple via a carrier-mediated mechanism. Electrically controlling the magnetic properties of doped TIs also holds great importance for future device applications. For example, by gate tuning the carrier density, the coercivity or AHE may be modulated^18,19^. The use of electrolyte gating has also been demonstrated in undoped TIs^20^, while it has not been applied to magnetically doped TIs. Recently, we characterized Cr_x_Sb_2−x_Te_3_ magnetic topological insulator (MTI) samples using x-ray magnetic circular dichroism (XMCD) and observed carrier mediated coupling^21^. In addition, we also reported that a good crystalline order is maintained in molecular beam epitaxy (MBE) grown ~60 quintuple layer (QL) thick films of Cr_x_Sb_2−x_Te_3_ (*x* = 0.15, 0.26, 0.42) using x-ray diffraction (XRD) and atomic force microscopy^13^. In this study, we present a comprehensive comparison of the variation in ferromagnetism in highly doped Cr_x_Sb_2−x_Te_3_ films (*x* ranging from 0.15 to as high as 0.76) through gated electrical transport, magneto-optical Kerr measurements and terahertz time domain spectroscopy. We conclude that the ferromagnetic order in the films is via the carrier-mediated RKKY mechanism.

## Results and Discussion

**Figure 1:**
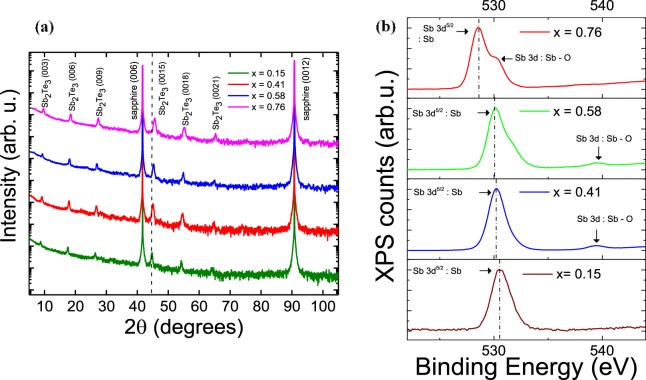
(**a**) X-ray diffraction spectra (2θ) of the Cr-doped Sb_2_Te_3_ films on *c*-plane sapphire substrates [Al_2_O_3_ (0001)] as a function of Cr concentration *x*. The (0 0 *l*) film peaks (*l* = 3, 6, …) are labelled. The dashed vertical line indicates the position of the peak for the lowest doping concentration, and a shift of the peak position to larger angles (smaller *c*-axis lattice constants) is found with increasing doping concentration. (**b**) XPS spectra obtained from the 3*d* Sb shell. Upon increasing the Cr concentration *x*, the peaks shift towards lower energy indicating the doping of Cr into Sb sites.

Cr-doped Sb_2_Te_3_ thin films with a typical thickness of 20 nm were grown by MBE in an ultra-high vacuum chamber (UHV) with a base pressure lower than 2 × 10^−10^ Torr using a two-step growth process described in the Methods section. X-ray diffraction (XRD) was carried out to confirm their tetradymite-type crystal structure, shown in the Fig. 1(a). All samples display the (0 0*l*) peaks (*l* = 3, 6, 9, …), confirming that the films grow along the *c*-axis direction. After comparing possible characteristic diffraction peaks from pure Sb, Te and their other compounds such as SbTe, SbTe_2_ and Sb_2_Te_5_, no traces of any secondary phase formation were found in any of the samples, up to and including the highest doped sample (*x* = 0.76). For increasing Cr doping concentration, a reduction in the *c*-axis lattice parameter from 30.393 Å in a sample with *x* = 0.15 to 30.177 Å (*x* = 0.41), 30.057 Å (*x* = 0.58) and 29.834 Å (*x* = 0.76) for the highest doped samples was observed, marked by a shift in the (0 0*l*) peaks (dotted line), consistent with a substitutional doping scenario. XPS measurements were performed to explore the effect of Cr doping on the bonding properties. For the XPS analysis the samples were prepared in a similar manner as for electrical and optical measurements discussed below. Figure 1(b) shows the shift in the spectra of the Sb 3*d* peaks upon introducing the Cr dopant. In pristine sample of Sb_2_Te_3_ the Sb 3*d* peak is expected at 528–529 eV. A small shift to 530.5 eV in the present case for the sample with the lowest doping concentration (*x* = 0.15) can be attributed to surface oxidation (labelled with arrows). Upon increasing the Cr concentration, the peaks shift towards the un-oxidized, pristine value of 528 eV, thus indicating that the doping with Cr prevents further oxidation of Sb_2_Te_3_, consistent with earlier reports^11,22^. In other words, Cr atoms are more prone to oxidation compared to the Sb atoms, and upon substitution, the degree of surface oxidation of the sample is reduced. This conceivably demonstrates that Cr atoms are substituting the Sb atoms in the structure. Correspondingly, upon fitting the peaks, we observe that it is possible to change the doping levels in Cr_x_Sb_2−x_Te_3_ from *x* = 0.15 to *x* = 0.76. A negligible shift in the XPS pattern of Te 3*d* (Supplementary Figure S1) is observed upon increasing the dopant concentration for the peaks at 573, 583 eV (Te 3*d*), and 575, 586 eV (Te-O). Moreover, it is difficult to ascertain the substitution of the Cr at Te sites due to the fact that the peaks corresponding to the Te (3*d*) and Cr (2*p*) binding energies closely resemble each other. Nevertheless, we have demonstrated in our previous work that Cr substitutes an ion of a smaller ionic radius, i.e., Sb^13^.

**Figure 2:**
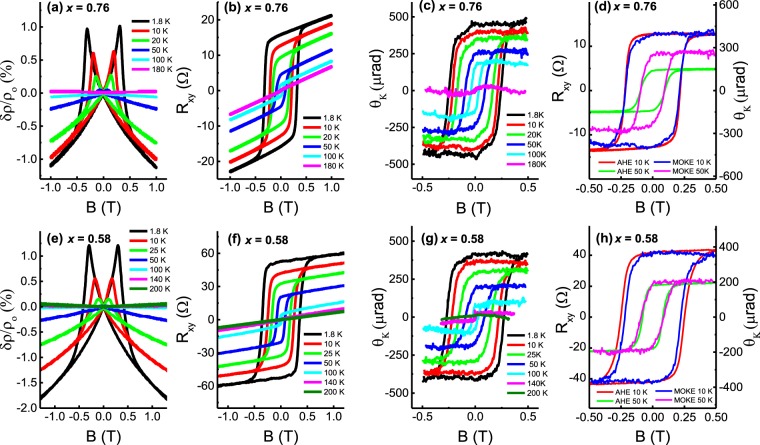
(**a**,**e**) Temperature-dependent negative magnetoresistance ratio for 20-nm-thick samples with *x* = 0.76 and 0.58 caused by spin dependent scattering of carriers. (**b**,**f**) Hall resistance *R*_*xy*_ in samples with *x* = 0.76 and 0.58 showing hysteretic behaviour, corresponding to the anomalous Hall effect at different temperatures from 1.8 K to 180 K. (**c**,**g**) Temperature dependent Kerr angle *θ*_*K*_ as a function of field for *x* = 0.76 and 0.58. (**d**,**h**) Magnetic hysteresis loops comparing the anomalous Hall resistance *R*_*xy*_ and Kerr angle *θ*_*K*_ as a function of magnetic field in a film with *x* = 0.76 and *x* = 0.58 at 10 K and 50 K. Both measurements are in good agreement with each other.

We used magneto-transport and magneto-optical measurements down to 1.8 K to characterize the ferromagnetic ordering in a series of samples with progressively increasing Cr doping, *x* from 0.15 to 0.76. Hall traces show that all Cr_x_Sb_2−x_Te_3_ films have hole-type carriers and display a similar trend, hence we take the highest doped samples with *x* = 0.76 and 0.58 as examples and all further data presented here are recorded for these samples unless specified otherwise. Figure 1(b,f and c,g) shows the evolution of the Hall resistance, *R*_*xy*_, and polar Kerr angle, *θ*_*K*_, respectively, as a function of temperature and magnetic field, *B*. Clear, long-range ferromagnetic ordering indicating the easy axis of orientation pointing out-of-plane is observed in both MOKE and transport measurements. We also detected that the coercive field, *H*_*c*_, and the saturated Hall resistance at *B* = 0 were higher for the sample with *x* = 0.58 than for *x* = 0.76 although its Curie temperature, *T*_*c*_, was lower. Furthermore, we studied the Hall transport, *R*_*xy*_, with different directions of the magnetic field, *B*, with respect to the sample as shown in Supplementary Figure S3.1. As the angle *θ* of the sample changes from out-of-plane (*θ* = 90°) to in-plane (*θ* = 0°), the value of *R*_*xy*_ reduces and the slope gradually changes from positive to negative until we see a parabolic dependence at *θ* = 0°. This parabolic behaviour at *θ* = 0° indicates a strong out-of-plane spontaneous magnetization at zero field, confirming that the magnetic moments of the sample prefer to be in the perpendicular direction. This feature may be favourable for spintronic applications, where perpendicular magnetic anisotropy is more common than in plane magnetism^18,23^. Figure 1(a,e) illustrate the *B*-dependent magnetoresistance (MR) ratio $\frac{\delta \rho }{{\rho }_{0}}\,=\frac{{\rho }_{xx}(B)-{\rho }_{xx}(0)}{{\rho }_{xx}(0)}$ measured from 180 K to 1.8 K. The butterfly shaped hysteresis characterizes the negative MR caused by spin dependent scattering of carriers^24^ as local magnetic ordering comes into play at *T*_*c*_. The distance between the two peaks for all temperatures corresponds to the coercive field *H*_*c*_. *T*_*c*_ can be estimated as the temperature when *H*_*c*_ becomes zero. At this point, the long-range ordering disappears and a positive *MR* is observed corresponding the weak anti-localization (WAL) commonly observed in pristine TI samples^25,26^. An undoped 20-nm-thick Sb_2_Te_3_ sample was also measured which showed WAL [see Figure S3.2(a) of the Supplementary Information]. The WAL phenomenon demonstrates both the Dirac nature of the surface state carriers as well as the strong spin−orbit interaction in pristine TI materials^27^. On doping the Sb_2_Te_3_ film with Cr, the WAL response disappears and instead, an increase in the resistance at low fields corresponding to the MR effect is observed [Figure S3.2(b) of the Supplementary Information]. Figure 2(d,h) directly compares the MOKE signal, *θ*_*K*_, and the AHE signal, *R*_*xy*_, at 10 K and 50 K for the two samples with *x* = 0.76 and 0.58. The *H*_*c*_ values for both measurements are in good agreement while an anomaly is seen in *R*_*xy*_ and *θ*_*K*_ at 50 K for *x* = 0.76 [Fig. 2(d)].

**Figure 3:**
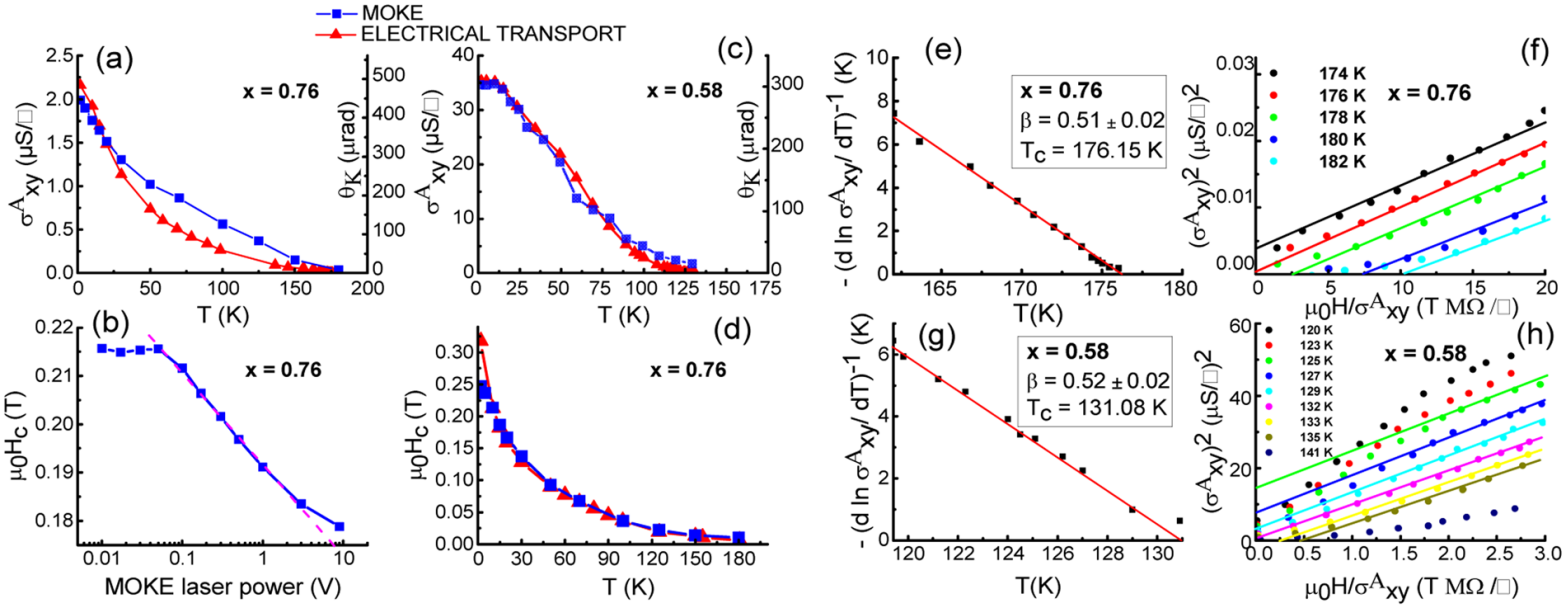
(**a**,**c**) Zero-field anomalous Hall conductivity (${\sigma }_{xy}^{A}$) and optical Kerr rotation (*θ*_*K*_) for samples with *x* = 0.76 and *x* = 0.58 as a function of *T*, respectively. (**b**) Coercivity (*H*_*c*_) for the *x* = 0.76 film extracted from MOKE hysteresis loop measurements as a function of laser power. The photovoltage is proportional to the light intensity incident on the film (10 V corresponds to ~10 mW). (**d**) Dependence of *H*_*c*_ with temperature for the film with *x* = 0.76. (**e**,**g**) Kouvel-Fisher plots showing the obtained values for *β* and *T*_*C*_ from the slope and intercept, respectively. (**f**,**h**) Arrott plots giving a *T*_*C*_ of 176 K (*x* = 0.76) and 132 K (*x* = 0.58).

To understand this further we studied the trend comparing the anomalous Hall conductivity, ${\sigma }_{xy}^{A}$, and Kerr rotation, *θ*_*K*_, as a function of temperature for both samples as shown in Fig. 3(a,c). Both curves are strikingly concave over a broad range of temperatures, similar to the data reported by Chang *et al*.^18^ in their transport data, but different from that of Zhou *et al*.^28^ in their SQUID data (both in Cr-doped TIs). Furthermore, the surface-favoured sensitivity of MOKE measurements might account for the discrepancy with transport measurements^29^, which probe the whole film. Magnetic properties of Cr-doped Sb_2_Te_3_ can also be affected by the laser illumination during MOKE measurements. When the laser is focused to a 3 μm spot size on the sample at *T* = 10 K, the coercivity decreases with increasing laser intensity as shown in Fig. 3(b). This relation fits well to a power law (dashed line) from 0.1 V to 3 V photovoltage, which corresponds to approximately 0.1 mW to 3 mW energy incident on the film. Locally induced heat from the laser is likely to account for this coercivity relation^30^. Below 0.05 mW laser power (0.05 V photovoltage) the coercivity plateaus at ~0.215 T, showing good agreement with AHE measurements at 10 K. This suggests, as compared to electrical transport measurements, that the laser power below 0.5 mW is unlikely to significantly affect magnetism in the film. Figure 3(d) shows the *H*_*c*_ dependence on temperature carried out at laser power = 0.5 mW. Both measurement sets are in good agreement with each other. Theoretically, *T*_*c*_ can be established as the point when ${\sigma }_{xy}^{A}$ and *H*_*c*_ both fall to zero at zero magnetic field, but in experiments, a tail is commonly observed above *T*_*c*_. This may be due to sample inhomogeneity or a finite remanent field arising from the superconducting magnetic coils in the cryostat^31^. Hence it is best to accurately determine *T*_*c*_ by using Kouvel-Fisher (K-F)^32^ and Arrott-Noakes^33^ plots.

Close to *T*_*c*_, all ferromagnetic material properties are determined by critical fluctuations following power law dependences^34^. In the case of K-F plots, the reduced magnetization follows $\,{(\frac{dlnM}{dT})}^{-1}=-\,\frac{1}{\beta }({T}_{c}-T)$, where *β* is the fitting coefficient. Replacing *M* with ${\sigma }_{xy}^{A}$ and plotting ${(d\mathrm{ln}{\sigma }_{xy}^{A}/dT)}^{-1}$ against *T* yields *β* from the slope, and the value of *T*_*c*_ from the intercept on the *T* axis. Figure 3(e,g) shows the K-F plots with linear fitting performed over the reduced temperature ranges for the samples with *x* = 0.76 and 0.58, giving a value of *β* = 0.5 1 ± 0.02 and 0.52 ± 0.04 for both samples. These values are consistent with the value of 0.5 predicted in the mean-field model. The corresponding *T*_*c*_ values are (176 0.46 ± 0.02) K and (131.51 ± 0.02) K. In the case of Arrott- Noakes plots, the equation takes the form: ${(\frac{h}{{\sigma }_{xy}^{A}})}^{1/\gamma }={\rm{a}}t+b{({\sigma }_{xy}^{A})}^{1/\beta }$, where *a* and *b* are assumed to be temperature-independent coefficients related to the critical amplitudes, while *β* and *γ* are the implicit critical exponents. Figure 3(f,h) show modified Arrott plots using the mean field exponents values of *β* = 0.5 (deduced from K-F plots) and *γ* = 1 for the samples with *x* = 0.76 and 0.58. The plot gives parallel straight lines for each *T*, with that for *T* = *T*_*c*_ passing through the origin. The corresponding *T*_*c*_ values obtained are 176 K and 132 K and hence justify the use of the mean field critical exponent value.

**Figure 4:**
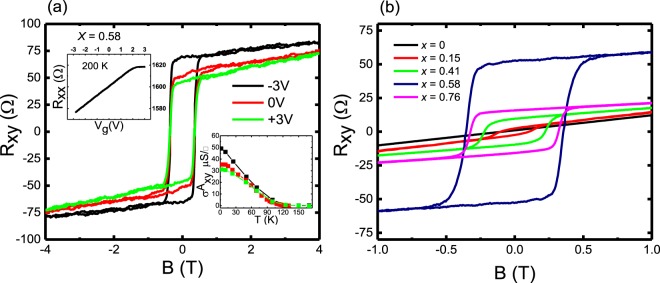
(**a**) Gate bias dependence of the Hall traces *R*_*xy*_ in a sample with *x* = 0.58 at 1.8 K showing an increase in the AHE from −3 V to +3 V. Top inset: *R*_*xx*_ vs *V*_*g*_ curve at 200 K. Lower inset: *T* dependence of the anomalous Hall conductivity $\,{\sigma }_{xy}^{A}$ for *V*_*g*_ = −3 V, 0 V and +3 V. (**b**) Hall resistance *R*_*xy*_ at 1.8 K for various Cr doping *x*.

Applying an ion gel as a gate dielectric allows for the efficient control of the carrier densities at very low operating voltages, making it superior compared to other gating methods which suffer from high leakage currents, slow polarization responses, and limited transistor operation speeds at less than 100 Hz^35,36^. A DEME-TFSI based ion gel was used (we refer the reader to the methods section on device fabrication and electrical transport for further details). By applying a positive gate voltage, the DEME^+^ cations accumulate on the flat surface of the Hall bar channel and an electric double layer which acts as a capacitor with nanoscale thickness forms at the liquid/solid interface. Figure 4(a) shows the Hall resistance *R*_*xy*_ modulation with gate bias fixed at −3 V, 0 V and +3 V at 200 K for a sample with *x* = 0.58 cooled to 1.8 K. The amplitude of the hysteresis loop increases, showing an increase in the AHE as the gate bias is lowered from +3 V to −3 V. The leakage current in the ion gel dielectric was less than 100 nA. The total carrier density *p* changes from 1.89 × 10^14^ cm^−2^ at −3 V to 9.31 × 10^13^ cm^−2^ at +3 V. The inset on the top of Fig. 4(a) shows *R*_*xx*_ vs *V*_*g*_ sweep at 200 K (the temperature where gel modulation is possible before cooling to 1.8 K) displaying *R*_*xx*_ increasing as *V*_*g*_ increases. The bottom inset of Fig. 4(a) shows the temperature dependent ${\sigma }_{xy}^{A}$ deduced from the saturated behaviour at +3, 0 and −3 V. An increase in the ${\sigma }_{xy}^{A}$ at −3 V is explained by a larger hole concentration on applying an electric field, resulting in enhanced ferromagnetic properties (AHE), directly suggesting a hole-mediated ferromagnetic interaction among local Cr spins. Note that there is no apparent change in *T*_*c*_ with gate voltage. To check if the gate control of the magnetic properties is reproducible, the measurements were repeated in the opposite sequence, i.e., first +3 V, followed by 0 V, and then −3 V as shown in Supplementary Figure S4(b). The results were replicated within ~10%, which can be attributed to slow polarization relaxation of the gel^20^. In Fig. 4(b) we plot the total Hall resistance versus perpendicular magnetic field at 1.8 K for different Cr concentrations. The coercive field and saturated Hall resistance increase with Cr concentration, except for the sample with *x* = 0.76. A similar trend was also reported by Lee *et al*. in Mn doped Bi_2_Te_3_^37^, which they attributed to structural flaws arising from a Bi bilayer sandwiched between two QLs. Our XRD data shows no parasitic peaks [see Fig. 1(a)] over the entire scan range, even for the highest doped sample, where a reduction in the *c*-lattice parameter corresponds to Sb being replaced by Cr. The homogeneity of magnetic ordering in transition metal doped TIs has been under debate and a few possible scenarios are proposed such as super-paramagnetism^38^, long-range ferromagnetism, antiferromagnetism^39^ and cluster formation^40^. These scenarios depend on the location and concentration of the Cr atoms in the crystal. For example, Cr atoms sitting at interstitial sites may form clusters and couple antiferromagnetically, while Cr replacing Sb coupled ferromagnetically^41^. Our results may perhaps be explained by the spontaneous coexistence of ferro- and antiferromagnetic coupling in the highest doped sample leading to a lower saturation resistance at 0 T for *x* = 0.76. In order to further our understanding and elucidate the mechanism of magnetism, a detailed study was carried out to comprehend the relationship between carrier densities, *T*_*c*_, and Cr concentration trends using terahertz time domain spectroscopy (THz-TDS).

**Figure 5:**
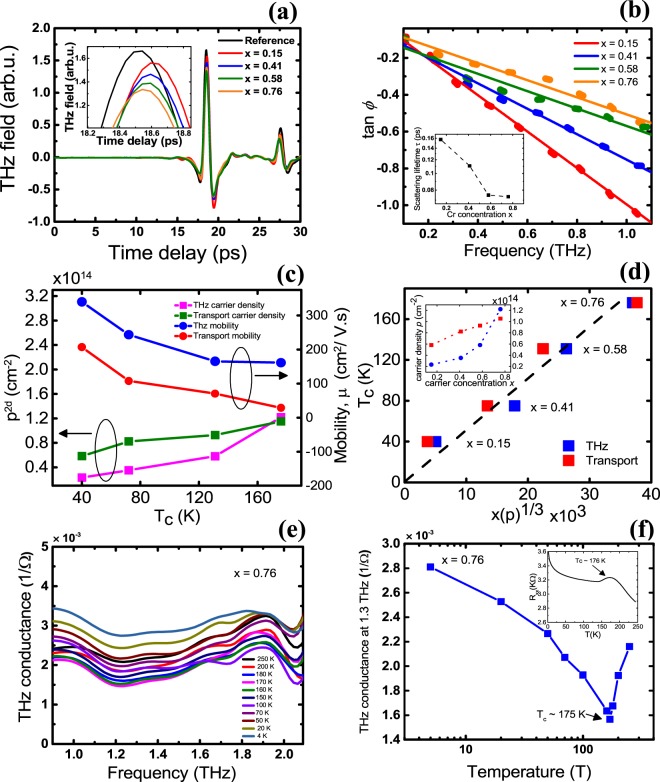
(**a**) Time domain picosecond pulse response transmitted through 20 QL Cr_x_Sb_2−x_Te_3_ films with varying Cr concentrations *x*. The inset shows a magnified pulse response transmitted through the Cr_x_Sb_2−x_Te_3_ film and a reference transmission through a (0001) sapphire substrate at 4 K. (**b**) Plot of tan *ϕ* vs frequency. The solid lines represent the linear fit to each data set. The inset shows the scattering lifetime *τ* vs x. (**c**) The plots of mobility *μ* and carrier density *p* vs Curie temperature *Tc* deduced from the THz measurements (blue and pink curves) and Hall measurements (red and green curves), respectively. (**d**) Sheet carrier density, *p*^*1/3*^ *x*, as a function of *T*_c_ obtained from the THz measurements (blue curve) and Hall measurements (red curve). The inset shows carrier density *p* vs carrier concentration *x*. (**e**) THz conductance spectra of Cr_0.76_Sb_1.24_Te_3_ showing distinct features at ~1.9 THz referring to the optical phonon. (**f**) THz conductance at 1.3 THz as a function of *T*.

It is well-known that device processing makes it more challenging to probe the surface states of pristine TI thin films^42^, as TIs are very sensitive to water and organic solvents. To circumvent this issue, we employed contact-free and non-invasive THz–TDS to study optical conductivities and determine the carrier densities and mobilities for all the samples, and compare them directly with the results from electrical transport measurements. The carrier damping rate in TIs (typically~10^−13^ s^−1^) lies in the THz frequency range (0.1–2.2 THz), making broadband THz spectroscopy a particularly sensitive probe of TI carrier dynamics. Moreover, the energies typical of collective quasiparticles such as optical phonons, have a fingerprint across the energetic range of the THz radiation (0.4–4 meV) making it a powerful technique to study TIs^27^. The time-resolved THz transmission through all Cr_x_Sb_2−x_Te_3_ thin films was measured at 4 K as shown in Fig. 5(a). The primary transmitted THz field intensity shows a systematic reduction with increasing Cr concentration *x* [see inset in Fig. 5(a)] which signifies an increased free carrier absorption in the film. To further quantify this behaviour, we extracted the carrier densities of each sample.

Using the Tinkham’s equation for thin films^43^ and substituting for the complex Drude conductivity as $\tilde{\sigma }(\omega )={\sigma }_{0}/(1-i\omega \tau )$, we obtain the following relationship between the imaginary and the real parts [see Figure S5.1(a,b) of the Supplementary Information] of the transmission coefficient: $Im.\,\{\tilde{T}(\omega )\}/Re.\,\{\tilde{T}(\omega )\}=$ $-\,\omega \tau =\,\tan \,\varphi$, where *ϕ* is the phase angle between the sample and substrate waveforms, *ω* is the angular frequency and *τ* is the scattering lifetime for the carriers. Plotting tan *ϕ* as a function of frequency [Fig. 5(b)], and fitting it with a linear regression, the slope yields the scattering lifetime *τ* [inset to Fig. 5(b)]. Furthermore, the scattering lifetime τ, obtained from THz-TDS, was used to calculate the THz mobility using μ = τe/m*(assuming the bulk hole effective mass m* in Sb_2_Te_3_ is 0.78 m_e_, with m_e_ as the electron rest mass), as shown in Fig. 5(c).The carrier concentration *p* can then be obtained using the following relationship between sheet conductance *G*_2*D*_ and *p*: *G*_2*D*_ = *G*(*ω* → 0) = *μep*. This yields the plot shown in Fig. 5(c), directly comparing the values of *p* and *μ* extrapolated from the slope of *R*_*xy*_ vs *B* at high fields [from Fig. 4(b)]. The carrier densities (and mobilities) in both measurements increase (decrease) roughly linearly with the Curie temperature *T*_*c*_ [*T*_*c*_ obtained from Fig. 3(f,h)] yielding the carrier dependence of the magnetic ordering temperature. It is interesting to note that the values of *p* obtained from the THz measurements tend to be smaller than those deduced from the Hall transport measurements. We used the bulk hole effective mass m* = 0.78 *m*_*e*_ in calculating the values for the THz measurements^44^. Due to the anisotropic nature of the upper and lower valence bands in Sb_2_Te_3_ the effective mass varies from m* = 0.034 *m*_*e*_ to m* = 1.24 *m*_*e*_^44,45^. This is likely to be one of the origins for the quantitative discrepancy in the parameters obtained from THz and transport measurements. Furthermore, Ar^+^ ion milling used during the Hall bar processing has been reported to increase the transport carrier density in topological insulators^46^. Figure 5(d) shows the relationship between *T*_*c*_ and *xp*^*1/3*^. In the case when magnetism is mediated through carriers in the mean field approximation, the Curie temperature is given by^39^: ${T}_{c}=\frac{S(S+1)}{3{k}_{B}}n{J}^{2}\chi$, where S is the spin quantum number of Cr, *n* is the number density of Cr in the material, *J* is the exchange interaction constant between localized Cr spin and itinerant holes, and *χ* is susceptibility of itinerant hole spins. In the above equation, *χ* is characterized as: $=\frac{{m}^{\ast }}{{h}^{2}}{k}_{F}=\frac{{m}^{\ast }}{{h}^{2}}{(3{\pi }^{2}p)}^{\frac{1}{3}}$, where m* is hole effective mass, *h* is Planck constant, and *p* is hole density. The above two equations indicate that in mean field theory, *T*_*c*_ will be proportional to *xp*^*1/3*^. We plotted *T*_*c*_ with respect to *xp*^*1/3*^ in all samples with results obtained from electrical transport and THz measurements [Fig. 5(d)]. Our results demonstrate a good proportionality between *T*_*c*_ and *xp*^*1/3*^, clearly supporting the validity of the mean field approximation in the parabolic band model, and strongly suggesting RKKY mediated magnetism in our Cr_x_Sb_2−x_Te_3_ films. This behaviour is similar to that reported by Zhou *et al*.^28^ and Li *et al*.^47^. The inset of Fig. 5(d) illustrates the proportionality between the Cr concentration *x* and the carrier density *p* with a monotonic increase in *p* with *x* also consistent with the mean field theory.

We further performed temperature dependent THz-TDS on the Cr_0.76_Sb_1.24_Te_3_ sample (largest Cr concentration) and deduced the corresponding THz conductance spectra [Fig. 5(e)], also see Supplementary Figure S5.2 for the time domain measurement. A detailed description of the method used to obtain the THz conductance is discussed elsewhere^27^. The THz conductance spectra in Fig. 5(e) shows a Drude type response with a characteristic phonon signature at 1.9 THz, consistent with previous reports of THz measurements on Sb_2_Te_3_ and other TIs^48,49^. Figure 5(f) shows the THz conductance response of Cr_0.76_Sb_1.24_Te_3_ plotted as a function of temperature. As the temperature is reduced from 300 K to 4 K, the THz conductance decreases rapidly indicating a freezing of the bulk carriers in the TI film. However, the THz conductance begins to gradually increase with a further reduction in temperature due to an insulator-to-metal transition at ~175 K. Such a behaviour can be attributed to the spin disorder scattering that sets in at the paramagnetic to ferromagnetic transition^50,51^. The temperature (~175 K) obtained from our THz measurements is in close agreement with the *T*_*c*_ value obtained from the transport measurements, and also matches the resistance vs temperature plot from transport shown in the inset of Fig. 5(f). Introducing a magnetic dopant in TI breaks time reversal symmetry^52^ and a bulk type insulator response is expected from the film (conductance decreasing with decreasing temperature). However below 175 K, the THz conductance shows a metallic response as shown in Fig. 5(f), indicating that the magnetism could be RKKY mediated. Table 1 summarizes all sample properties.

**Table 1:** Summary of the physical parameters of the Cr-doped Sb_2_Te_3_ thin films as determined by different methods. The Cr atomic concentration was measured and calculated using XPS, 2D carrier density (*p*), and mobility obtained from electrical transport and THz-TDS. *T*_*c*_ was deduced from electric transport.

Sample	*p* transport (10^13^ cm^−2^)	*p* Thz (10^13^ cm^−2^)	*µ* transport (cm^2^/Vs)	*µ* Thz (cm^2^/Vs)	*T*_c_ (K)
Cr_0.15_Sb_1.85_Te_3_	5.83	2.33	207	340	35
Cr_0.41_Sb_1.59_Te_3_	8.24	3.52	107	243	74
Cr_0.58_Sb_1.42_Te_3_	9.28	5.81	70	165	132
Cr_0.76_Sb_1.24_Te_3_	11.52	12.20	28	161	176

In conclusion, we have carried out MBE growth, structural characterization by XRD, and electrical and optical measurements on magnetically doped Cr_x_Sb_2−x_Te_3_ TI thin films using XPS, electric transport, MOKE, and THz-TDS. On increasing the Cr doping concentration, the crystal structure remains intact up to *x* = 0.76 with Cr being incorporated on Sb sites. AHE and MOKE measurements plotted as a function of magnetic field and temperature confirm out-of-plane ferromagnetic ordering and are consistent with each other. We measured a *T*_c_ of 176 K for the highest doped sample and employed ion gel gating on a sample with *x* = 0.58 to vary the chemical potential. We observed an increase in the magnitude of the AHE as the level of hole doping was increased by up to 50% with a gate bias variation of only ±3 V, which corresponded to an increase in carrier density by 50%. This directly suggests a strong correlation between carrier concentration and ferromagnetism originating from RKKY interaction. On plotting carrier densities and mobilities for all Cr doped samples obtained entirely from optical THz-TDS, and comparing them directly to transport measurements, we found that the *T*_*c*_ obtained from transport was proportional to *xp*^*1/3*^, further supporting a mean field approximated RKKY interaction scenario. Our work presents a unique comparison of optical measurements using THz-TDS and MOKE, and electrical transport measurements to achieve a complete characterization of Cr doping in Sb_2_Te_3_ thin films and their ferromagnetic properties. Moreover, these results provide a new pathway to explore quantum phenomena based on the magnetically induced gap opening at the Dirac point in topological insulators.

## Methods

### MBE growth of Cr-doped Sb_2_Te_3_

High purity Sb (6N) and Te (6N) were co-evaporated from standard effusion cells along with a high temperature cell for Cr (5N) to obtain high quality stoichiometric Cr-doped Sb_2_Te_3_. We started with a 5-nm-thick, undoped Sb_2_Te_3_ seed layer (see ref.^13^ for details). The flux ratio of Te per (Sb + Cr) was kept at ~10:1. The Te overpressure ensures reduction in vacancies commonly observed in this material system^53^. The Cr doping concentration was precisely controlled by adjusting the cell temperature to grow the next 15 nm of Cr-doped Sb_2_Te_3_. It was found that the Cr concentrations follow approximately the measured beam flux monitor fluxes. The typical growth rate for our experiment was ~1 quintuple layer/minute (QL/min). Finally, we grew a 3-nm-thick amorphous Te capping layer at room temperature prior to taking the sample out of ultra-high vacuum to avoid contamination.

### XPS

X-ray photoelectron spectroscopy (XPS) was measured using a Kα (Thermo Fisher Scientific) x-ray photoelectron spectrometer, equipped with a monochromatic Al Kα source. All XPS spectra were fitted by Gaussian/Lorentzian convolution functions with simultaneous optimization of the background parameters. The background was modelled using a combination of a Shirley and a Tougaard background.

### Device fabrication and electrical transport

After removing the films from the UHV chamber, 5 mm × 5 mm chips were cleaved in a clean room for device fabrication. Micro meter sized Hall bar geometries of dimensions *L* = 800 µm and *W* = 200 µm were produced, schematically shown in Figure S4(a) of the Supplementary Information. Standard S1813 photoresist was first spin coated onto the sample, followed by an optical lithography process using a mask pattern and an aligner. The mesa was then defined using Ar ion milling in a vacuum chamber with a calibrated rate of ~100 Å/min. 20/80 nm of Ti/Au were then evaporated as Ohmic contacts for the Hall bar in a thermal evaporator, followed by a standard lift-off process with acetone and isopropanol. The chips were then glued to chip carriers and Au wires were used to bond the contacts onto the pads using a ball bonder. Commercially purchased ionic-liquid *N*, *N*-diethyl-*N*-(2-methoxyethyl)-*N*-methylammonium bis (trifluoromethylsulphonyl-imide) (DEME-TFSI) was then drop-casted carefully using a micropipette on top of the channel of the Hall bar as shown in Figure S4(b) of the Supplementary Information. The samples were screwed to a probe and dipped into a continuous flow cryostat with a base temperature of 1.8 K equipped with a 9 T superconducting magnet. The Hall, *R*_*xy*_, and longitudinal, *R*_*xx*_, resistances were measured using a standard AC lock-in four-terminal method with the current applied in-plane (along *x*) and the magnetic field perpendicular to the film. All measurements were carried out with an excitation current of 1 µA at a frequency of 77 Hz. For gating measurements, the ion gel was biased at 200 K to fix the voltage at +3 V or −3 V, before cooling the gel along with the sample to 1.8 K.

### MOKE

The magnetic properties of all Cr-doped Sb_2_Te_3_ thin films were characterized by magneto-optic polar Kerr effect (MOKE) measurements. The MOKE describes the polarization change of an electromagnetic wave reflected from the surface of a magnetic material. When a linearly polarized laser is incident and reflected perpendicular to the sample surface, i.e., polar MOKE, the change of polarization degree will be proportional (to first order) to the material’s out-of-plane magnetization. The optical path of the polar MOKE experiment is illustrated in Supplementary Figure S2. The sample was mounted on a piezoelectric XYZ stage with 10 nm step size, in the same cryostat as used for the transport measurements. The linearly polarized laser beam from an 850-nm diode laser was focused down to 3 μm diameter onto the sample via an objective lens, and reflected back into the same path. Modulation at 100 kHz was achieved by passing light through a photoelastic modulator (PEM). To minimize unwanted contribution from photoexcitation and room lighting, an 850-nm bandpass filter was placed in front of the photodetector. The Kerr rotation angle^54^ can be calculated as: ${\theta }_{K}=\frac{\surd 2}{4{J}_{2}(A)}\frac{{V}_{AC}}{{V}_{DC}}$, where *J*_2_*(A)* is the first kind Bessel function, *A* is the PEM retardation angle (kept at *π*), *V*_*AC*_ is the photovoltage at 100 kHz due to the PEM modulation, and *V*_*DC*_ is the DC photovoltage.

### Terahertz time domain spectroscopy

Broadband terahertz time domain spectroscopy was carried out on all samples with different Cr concentrations, using a Tera K15-T-Light MENLO system. A 60 mW pump laser with 90 fs pulse duration at 1560 nm (repetition rate of 100 MHz) was split into two beam paths: (i) first the beam was focused down to a 40 μm spot onto the THz emitter, resulting in a broadband THz emission with a spot size of ~1 mm, and (ii) the second beam went through the delay stage for THz detection. The temperature dependent measurements were carried out using a Janis continuous flow cryostat with optical access capable of reaching a base temperature of 4 K. In the ultrathin-film limit, THz conductance spectra were obtained from the normalized transmission using Tinkham’s theory^43^. A detailed account of the optical setup and measurement is described elsewhere^27^.

## Electronic supplementary material

Supplimentary Information
