# Supplementary material for: Systematic Study of Ferromagnetism in CrxSb2−xTe3 Topological Insulator Thin Films using Electrical and Optical Techniques
Source: Sci Rep. 2018 Nov 19;8:17024. doi: 10.1038/s41598-018-35118-8 (PMC6242999; doi:10.1038/s41598-018-35118-8)
Supplement: Supplementary file 1 — Supplimentary Information [file 41598_2018_35118_MOESM1_ESM.docx]

Supplementary Online Material for

**Systematic Study of Ferromagnetism**

**in Cr_x_Sb_2-x_Te_3_ Topological Insulator Thin Films**

**using Electrical and Optical Techniques**

*Angadjit Singh^a,*^, Varun Kamboj^a^, Jieyi Liu^a^, Justin Llandro^a,c^, Liam B. Duffy^b^, Satyaprasad P. Senanayak**^a^, Harvey E. Beere^a^*, *Adrian Ionescu^a^, David A. Ritchie^a^, Thorsten Hesjedal^b,*^ and Crispin H.W. Barnes^a,*^*

*^a^Cavendish Laboratory, University of Cambridge, J. J. Thomson Avenue,*

*Cambridge CB3 0HE, United Kingdom*

*^b^Clarendon Laboratory, Department of Physics,University of Oxford, Oxford OX1 3PU, United Kingdom*

^c^ *Laboratory for Nanoelectronics and Spintronics, Research Institute of Electrical Communication, Tohoku University, 2-1-1 Katahira, Aoba-ku, Sendai 980-8577, Japan*

Correspondence to: as2331@cam.ac.uk, chwb101@cam.ac.uk

**Keywords:** topological insulators, Cr-doped Sb_2_Te_3_, anomalous Hall effect, ion gel gating, THz spectroscopy, MOKE, RKKY interaction

**Supplementary Section S1: X-ray photoelectron spectroscopy (XPS)**

**Figure S1**. X-ray photoelectron spectroscopy (XPS) spectra in the Te 3*d* region as a function of Cr concentration *x*.

Figure S1 shows XPS data measured in the Te 3*d* region. In order to confirm that Cr is occupying Sb sites, and not Te sites, we analyze the Te 3*d* peaks for the highly doped samples with *x* = 0.58 and *x* = 0.76. When comparing these doping concentrations, we only observe a negligible shift of 0.4 eV of the peak position of the Te 3*d* peaks at ~573eV. This slight shift in the XPS peaks could be due to the relative distribution of the Te-O and Cr-O bonds inside the crystal structure. Nevertheless, it should be noted that the peaks at 576 eV and 586 eV are difficult to be discerned as Cr-O or Te-O as their binding energies are very similar.

**Supplementary Section 2: Magneto Optical Kerr Effect (MOKE) setup.**

**
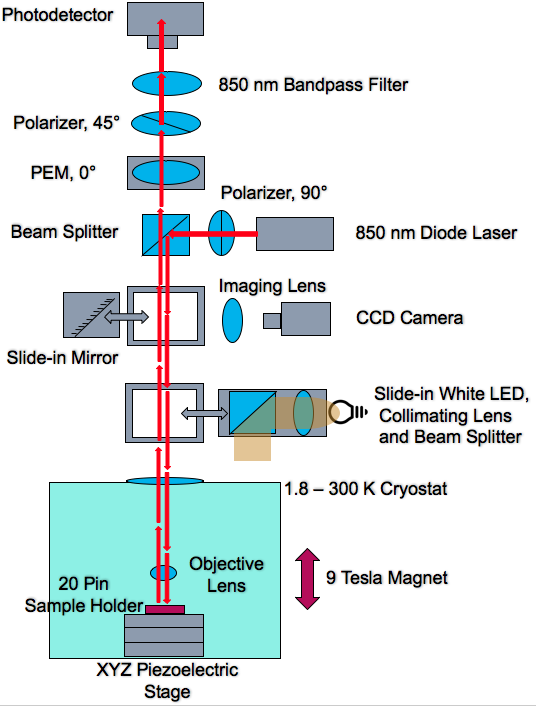
**

**Figure S2.** Schematics of the polar MOKE setup.

The light coming from an 850 nm diode laser was polarized by a Glan-Taylor polarizer and reflected by a beam splitter into the vertical main optical path. After passing through the cryostat window, the laser was focused down to a 3 μm spot on the sample via an objective lens. The then reflected light travelled through a PEM, a second Glan-Taylor polarizer, and an 850 nm bandpass filter before reaching the detector. A slide-in imaging system consisting of a CCD camera and a white illumination bulb is available if an optical image is to be captured.

**Supplementary Section 3.1: Angular-dependent Hall measurements on** **Cr_0.76_Sb_1.24_Te_3._**

I

θ

B

B’

**Figure S3.1 (a).** Angular dependence of the Hall resistance *R_xy_* at 1.8 K for a representative 20-QL-thick Cr_0.76_Sb_1.24_Te_3_ sample. The sketch on the right illustrates the definition of the out-of-plane angle θ with respect to the direction of the applied current I (θ = 0° along I).

Angular magneto-transport measurements were carried out on a 20-QL-thick Cr_0.76_Sb_1.24_Te_3_ sample as shown in Figure S3. As the angle *θ* of the sample changes from out-of-plane (*θ* = 90°) to in-plane (*θ* = 0°), the value of *R_xy_* reduces and the slope gradually changes from positive to negative until we see a parabolic dependence at *θ* = 0°. This parabolic behavior at *θ* = 0° indicates a strong spontaneous out-of-plane magnetization at zero field, confirming the out-of-plane magnetic anisotropy of the films.

**Supplementary Section 3.2:** **Magnetotransport measurements on an undoped, 20-nm-thick Sb_2_Te_3_ thin film topological insulator**

(a)

(b)

**Figure S3.2.** (a) Weak antilocalization effect in an undoped Sb_2_Te_3_ sample. Normalized conductivity changes as a function of magnetic field measured at 1.7 K. Each data set (dots) is fitted to the HLN formula. The cusp in conductance (or dip in resistance) is a characteristic feature of the WAL effect. (b) Normalized longitudinal resistance of a pure Sb_2_Te_3_ showing the WAL effect compared to a Cr doped Sb_2_Te_3_ sample with *x* = 0.15.

To check whether Sb_2_Te_3_ is a TI or not, we conducted magnetotransport measurements on a 20-nm-thick Sb_2_Te_3_ thin film grown on a sapphire substrate. Figure S3.2 (a) displays the normalised magneto-conductivity for the sample, defined as ∆$\sigma_{xx}= \sigma_{xx}\left( B \right)- \sigma_{xx}\left( 0 \right)$, at 1.7 K as a function of magnetic field *B*. A sharp increase in the conductivity is observed at low magnetic field *B*. This demonstrates the weak antilocalization (WAL) behaviour, a phenomenon which demonstrates both the Dirac nature of the surface state carriers as well as the strong spin−orbit interaction in pristine TI materials^25,26,27^.

The HLN equation describes the quantum correction to the conductivity due to localization or anti-localization effects as^27^:

∆$\sigma_{xx}= \sigma_{xx}\left( B \right)- \sigma_{xx}\left( 0 \right)= \alpha\frac{e^{2}}{2\pi^{2}\hbar}\left[ ln\left( \frac{\hbar}{4eBL_{\varphi}^{2}} \right)- \Psi\left( \frac{1}{2}+\frac{\hbar}{4eBL_{\varphi}^{2}} \right) \right]$ (1)

where $\Delta\sigma_{xx}$ indicates the normalized 2D sheet conductivity, *B* is the magnetic field perpendicular to the plane of the film, $e$ is the electron charge, $\hbar$ is the reduced Planck constant,$\Psi$ is the digamma function, $\alpha$ is the number of topological surface modes (TSM) and $L_{\varphi}$ is the phase coherence length.

We further obtained the value of the prefactor *α* by fitting the normalized conductvity data (dotted line) with the HLN model (solid line) described by equation 1 in Ref. [27] . A value of *α* ≈ 1 was obtained which strongly suggests a surface dominated transport with two symmetric decoupled channels (top and bottom), as expected in a pristine TI thin film^26^. When the samples were physically doped with Cr, instead of observing the WAL effect (a dip in resistance at 0 T or peak in conductance) as shown in Figure S3.2(a), we observed a peak in the resistance at ~ 0 T (or dip in conductance) corresponding to the magnetoresistance effect as shown in the Figure S3.2(b) for *x* = 0.15. A similar increase in the resistance was also observed with increasing *x* as displayed in Figure 2(a,e) of the manuscript.

**Supplementary Section 4: Electrostatic ion gel gating**

(b)

(a)

**Insulating Al_2_O_3_**

**substrate**

**ion gel DEME-TFSI**

S

D

G

**TI thin film Hall bar**

**V_xx_**

**V_xy_**

**Figure S4.** (a) Schematic of the ion gel drop casted on top of a Hall bar (b) Gate bias dependence of the Hall traces *R_xy_* in a sample with *x* = 0.58 at 1.8 K showing a decrease in the AHE from +3V to -3V.

To check if the controllability of the electrostatic gating was reproducible, the measurements presented in the main text were repeated on a sample with *x* = 0.58. The results were taken using the opposite sequence, i.e., first +3 V, followed by 0 V and then -3 V. The results are very similar to those reported in the main text, showing an increase in the anomalous Hall resistance at -3 V with decreased carrier density compared to +3V. This directly suggests carrier-mediated magnetism attributed to the RKKY mechanism.

**Supplementary Section 5.1:** **Real and imaginary transmission spectra through 20-QL-thick Cr_x_Sb_2-x_Te_3_ films.**

(a)

(b)

**Figure S5.1.** (a) Imaginary and (b) real transmission spectra as a function of frequency for different Cr concentrations.

Figure S5.1 shows the imaginary and real components of transmission, obtained from the Fourier transform of the time resolved primary pulse response [Figure 5(a) in the main text], through a 20-QL-thick Cr_x_Sb_2-x_Te_3_ film at 4 K. The imaginary part of the transmission signal reflects the accompanying phase changes in the real part of the transmission.

**Supplementary Section 5.2: THz response of the (0001)-oriented sapphire substrate and the 20-QL Cr_0.76_Sb_1.24_Te_3_ film.**

**(a)**

**(b)**

**Figure S5.2.** (a) Time-domain ps pulse response for transmission through a 20-QL Cr_0.76_Sb_1.24_Te_3_ film. The reference is transmission through the (0001) sapphire substrate. The inset shows a magnified time-domain ps pulse response for both cases. (b) Normalized transmission amplitude spectra with respect to the substrate (at the same temperature), obtained from the Fourier transform of the temporal THz signal through Cr_0.76_Sb_1.24_Te_3_ from 250 K to 5 K.

The time-resolved THz transmission through a 20-QL Cr_0.76_Sb_1.24_Te_3_ film was measured at various temperatures as shown in Figure S5.2(a). We further normalized the Fourier transform of the transmission through the Cr_0.76_Sb_1.24_Te_3_ film to the signal through the sapphire substrate at the same temperature, to remove any substrate effects [see Figure S5.2 (b)].
